# Harnessing the heart’s resistance to malignant tumors: cardiac-derived extracellular vesicles decrease fibrosarcoma growth and leukemia-related mortality in rodents

**DOI:** 10.18632/oncotarget.20454

**Published:** 2017-08-24

**Authors:** Lilian Grigorian-Shamagian, Soraya Fereydooni, Weixin Liu, Antonio Echavez, Eduardo Marbán

**Affiliations:** ^1^ Cedars-Sinai Heart Institute, Los Angeles, CA, United States of America; ^2^ Department of Biology, Stanford University, Stanford, CA, United States of America

**Keywords:** extracellular vesicles, cancer, fibrosarcoma, cardiosphere-derived cells, oncogenic safety

## Abstract

The heart is known for its resistance to cancer. Although different conjectures have been proposed to explain this phenomenon, none has been tested. We propose that the heart microenvironment may exert anti-cancer properties. So, our objective was to test the anti-oncogenic potential of cardiac-derived extracellular vesicles (EVs).

For that EVs secreted by cardiosphere-derived cells (CDCs, heart progenitor cells) were tested *in vitro* on fibrosarcoma HT1080. *In vivo* models comprised the xenograft HT1080 fibrosarcoma in athymic mice (n=35), and spontaneous acute lymphocyte leukemia in old rats (n=44). CDC-EVs were compared with two control groups: EVs secreted by bone-marrow derived mesenchymal stem cells (MSC-EVs) and phosphate-buffered saline (PBS).

Injection of CDC-EVs led to a 2.5-fold decrease of fibrosarcoma growth in mice (p<0.01 and p<0.05 for human and rat EVs, respectively) vs PBS group. The effect was associated with 2-fold decrease of tumor cells proliferation (p<0.001) and 1.5-fold increase of apoptosis (p<0.05) in CDC-EV vs PBS mice. Salutary changes in tumor gene and protein expression were observed in CDC-EV animals. CDC-EVs reduced tumor vascularization compared with PBS (p<0.05) and MSC-EVs (p<0.01). Moreover, CDC-EVs increased leukemia-free survival (p<0.05) in old rats vs PBS. MiR-146, highly enriched in CDC-EVs, may be implicated in part of the observed effects. In conclusion, this study presents the first evidence that ties together the long-recognized enigma of the “heart immunity to cancer” with an antioncogenic effect of heart-derived EVs. These findings open up cancer as a new therapeutic target for CDC-EVs.

## INTRODUCTION

The heart is known for its immunity to cancer. In contrast to other organs, the overall incidence of primary heart tumors was only 0.02% in an autopsy series in the United States, and only one-quarter of them were malignant [1]. Terminal differentiation and low turnover of cardiomyocytes were proposed as the main mechanisms of heart resistance to tumor formation [2]. However, this premise is brought into question by the facts that the heart contains a predominantly non-cardiomyocyte, proliferating population of fibroblasts, endothelial and vascular smooth muscle cells, which constitute ∼70% of all cells in the adult heart [3]. Moreover, the incidence of malignancies in the central nervous system [4], also characterized by a low rate of cell division, is much higher than that in heart. The unceasing and efficient oxygen-consuming metabolism of the heart, recently proposed as a potential anti-cancer mechanism [5], is challenged by the low incidence of cancer in many cardiac diseases related to altered oxygen supply-consumption balance (i.e. cyanotic congenital heart disease).

The concept of a tumor microenvironment has emerged as crucial during primary tumor formation, as well as in later stages of invasion and metastasis [6]. However, the hypothesis that the heart microenvironment may exert anti-cancer properties has not been tested so far. Cardiosphere-derived cells (CDCs) are heart progenitor cells in advanced pre-clinical and clinical development for regenerative medicine applications; CDCs have demonstrated efficacy in various cardiac pathologies and no safety-related issues to date [7–9]. The benefits of CDCs are mostly paracrine, mediated by nanoscale extracellular vesicles (EV), including exosomes [10], which may themselves turn out to be useful cell-free therapeutic candidates.

While trying to confirm the safety profile of CDC-derived EVs (CDC-EV) in an *in vivo* cancer model, we unexpectedly found a significant reduction of tumor growth. The role of EVs in cell-cell communication between tumor cells and surrounding cells has been highlighted as relevant to metastasis and tumor growth [11]. For these reasons, we evaluated the anti-oncogenic properties of CDC-EV and probed underlying mechanisms.

## RESULTS

### CDC-EVs decrease fibrosarcoma growth by decreasing proliferation and increasing apoptosis of tumor cells in mice

**Figure 1:**
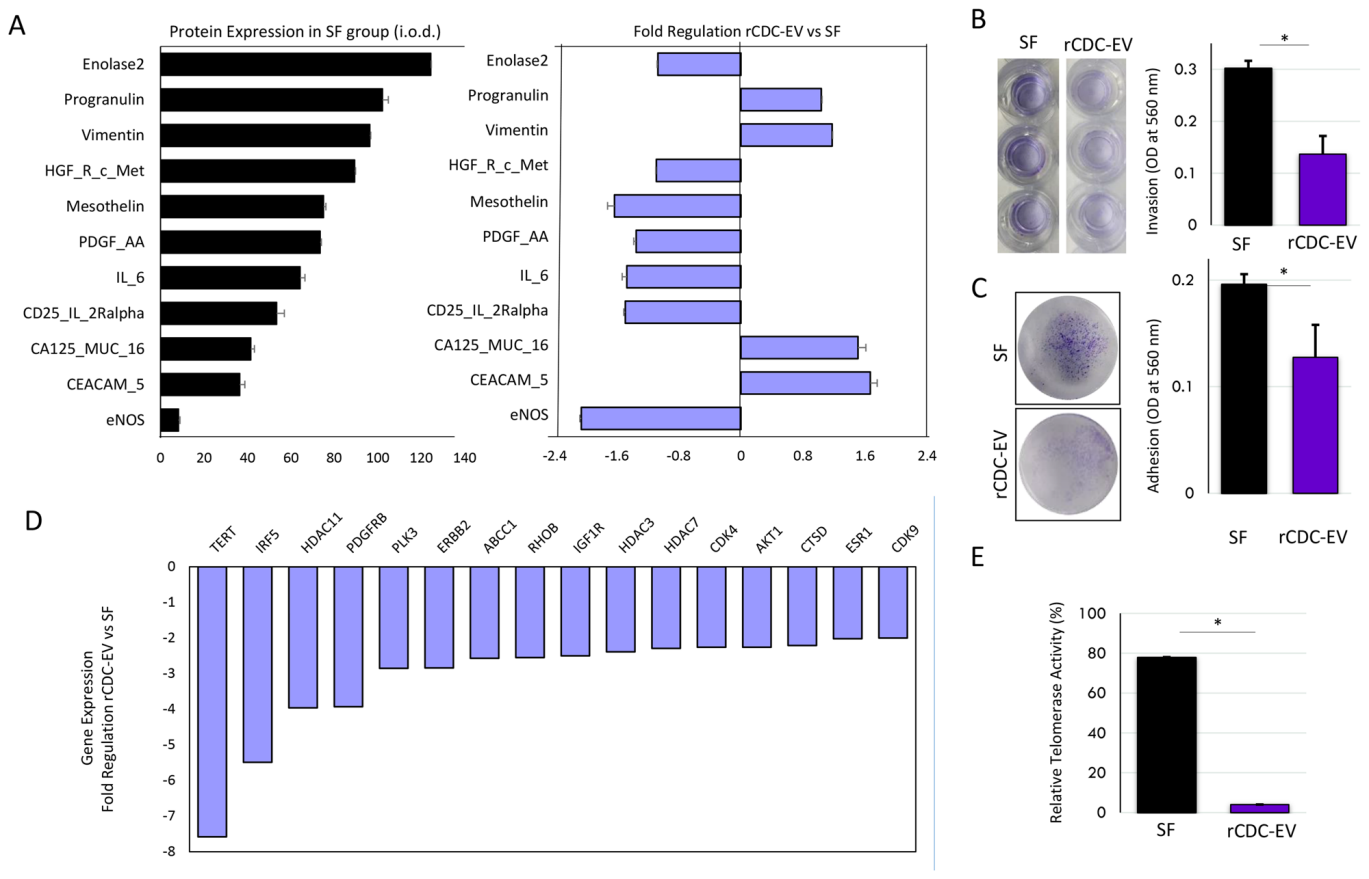
CDC-EVs negatively impact the aggressiveness of human HT1080 fibrosarcoma cells *in vitro* **(A)** Priming of HT1080 cells with CDC-EVs was associated with a favorably balanced modulation of cancer progression-related proteins compared to culture in serum-free media (SF) alone (lilac bars). Only significantly modulated (p<0.05) proteins are shown. Black bars represent the protein expression levels in the SF cells. **(B)** Representative wells and a decreased invasion capacity of HT1080 after priming the cells with CDC-EVs vs SF. **(C)** Representative wells and a decreased adhesion capacity of HT1080 after priming the cells with CDC-EVs vs SF. **(D)** Priming of HT1080 cells with CDC-EVs was associated with a down-regulation of many “cancer drug target” genes. Only significant (p<0.05) and more than two-fold modulated genes are shown. **(E)** Telomerase activity in extracts of HT1080 cells was determined following telomeric repeat amplification protocol in SF and CDC-EV primed cells, showing a marked decrease in the later group of cells. ^*^p<0.05. Bar graphs represent mean (±SEM). Minimum number of replicates per experiment was 3.

We characterized global CDC-EV-induced changes in expression of cancer-related proteins and genes using specific arrays, rather than focusing on a single pathway. After HT1080 cells were incubated for 96 hours with rat CDC-EVs or serum-free medium (SF) alone *in vitro* (Supplementary Figure 1A), significant differences were observed in 11 of 84 proteins analyzed (Figure 1A). Although not unidirectional, most of the observed differences (downregulation of proteins such as enolase 2, c-Met, mesothelin, PDGF-AA, eNOS, IL-6, and upregulation of CA125) suggested a negative impact of CDC-EVs on pathways associated with cancer. Negative effects were further confirmed as CDC-EV-primed HT1080 cells showed lower invasion and adhesion properties compared with cells incubated with SF medium alone (Figure 1B, C). Cancer drug target transcripts (n=84) were likewise quantified in HT1080 cells with or without CDC-EV priming. Thirty-five genes were significantly up or down-regulated (Supplementary Figure 1B); those with at least two-fold changes between groups are shown in Figure 1D. The most down-regulated was the TERT gene, coding for the catalytic subunit of telomerase. We further confirmed a marked decrease of telomerase enzymatic activity (Figure 1E) under the same conditions.

**Figure 2:**
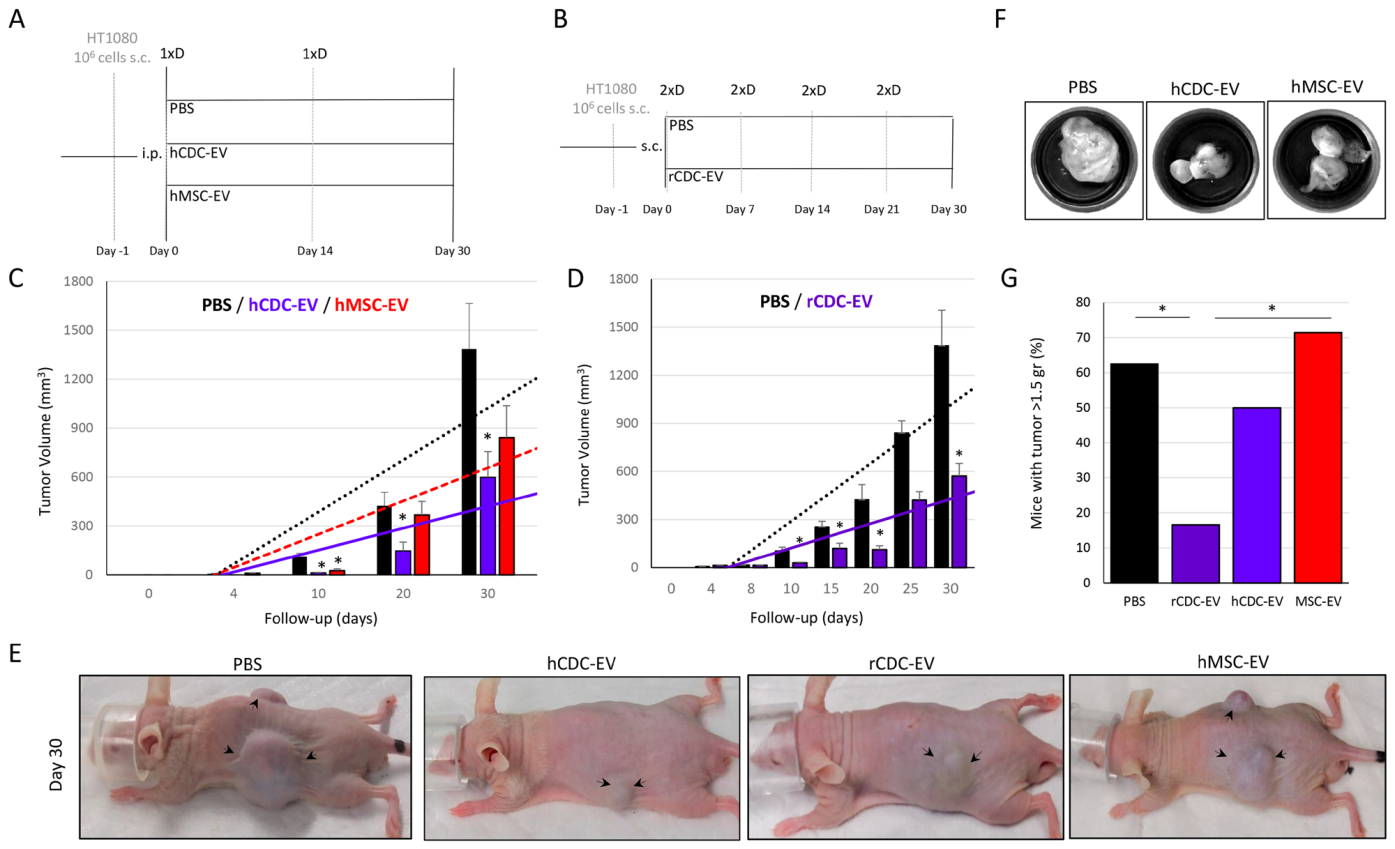
CDC-EVs decrease fibrosarcoma growth in mice **(A)** Study design where systemic (intraperitoneal –i.p.) delivery of human CDC-EVs (hCDC-EV; n=6) was compared to PBS (n=9) and human MSC-EVs (hMSC-EV; n=8) injections. 1xD refers to single dose. **(B)** Study design where local (subcutaneous, peritumoral – s.c.) delivery of rat CDC-EVs (rCDC-EV; n=6) was compared with local PBS (n=6) injections. 2xD refers to double dose. **(C)** External tumor growth measured with a caliper in the systemic-delivery protocol, showing a significant decrease in the hCDC-EV vs PBS groups. **(D)** External tumor growth measured with a caliper in the local-delivery protocol, showing a significant decrease in the rCDC-EV vs PBS groups. **(E)** Representative images of mice at day 30 with visibly smaller tumors (marked with arrows) in animals treated with human- and rat-CDC-EVs compared with the other two control groups (PBS and MSC-EV). **(F)** Representative images of the harvested tumors. **(G)** Bar graph showing the proportion of mice with the heaviest tumors (defined as tumor weigh more than the mean of 1.5 gr in all mice together). ^*^ p<0.05. Tumor growth’s bar graphs represent mean (±SEM).

**Figure 3:**
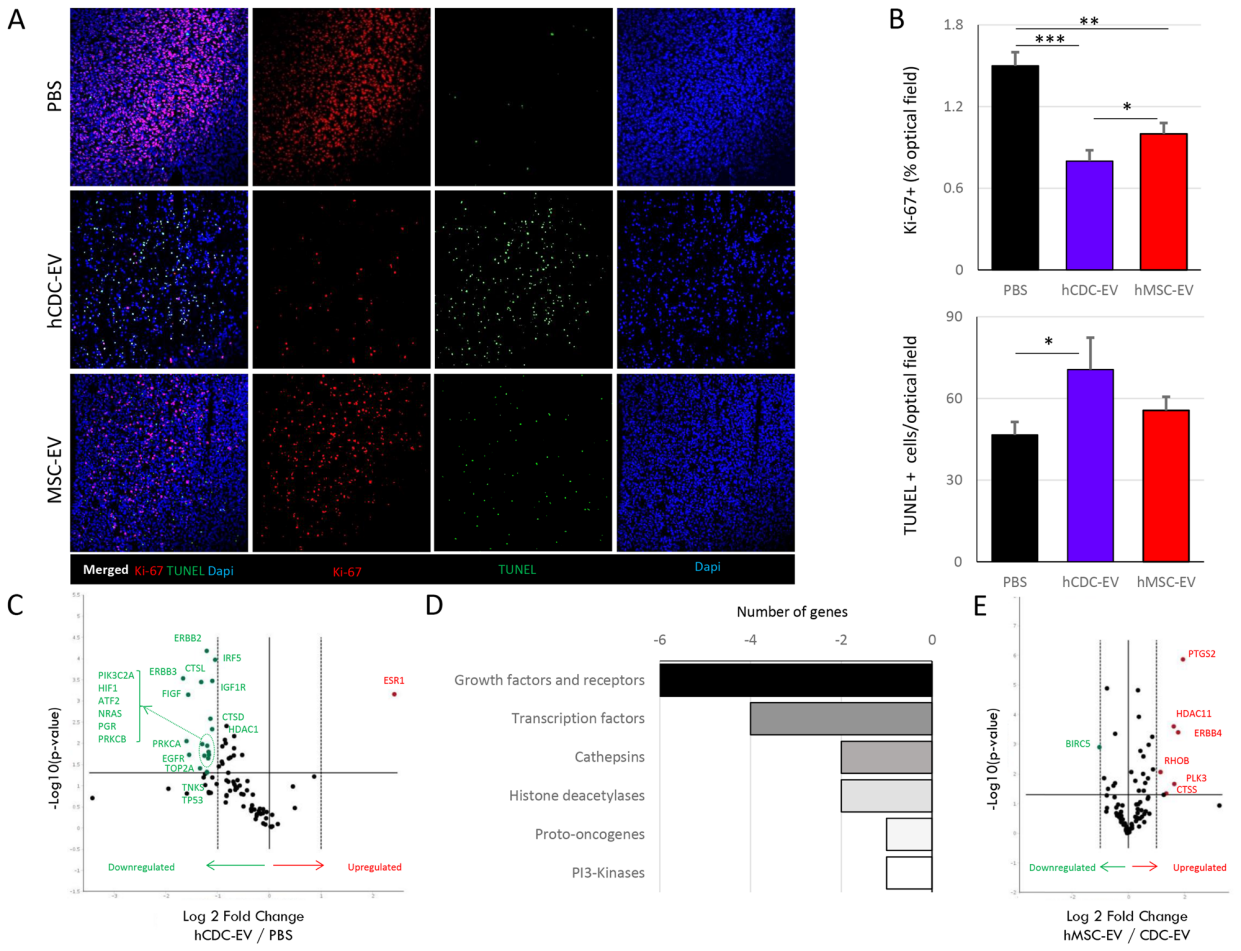
Local effects of the systemically delivered extracellular vesicles (EVs) at the tumor site in mice with fibrosarcoma **(A)** Immunostaining for Ki-67 and terminal deoxynucleotidyl transferase dUTP nick end labeling (TUNEL), showing a marked decrease of proliferation and increase of apoptosis in the CDC-EV treated (n=5) compared to PBS (n=5) injected mice. Differences in proliferation and apoptosis markers between CDC-EV and MSC-EV (n=6) treated mice were less marked. **(B)** Graphs showing differences in expression of Ki-67 (upper panel) and TUNEL (lower panel) between groups. **(C)** Volcano plot representing gene expression in CDC-EV vs PBS-treated mice. Genes with significant and higher than two-fold down- (green) and up-regulation (red) are referenced. **(D)** Schematic representation of the distribution of the highly (more than two-fold) down-regulated genes in CDC-EV vs PBS groups. **(E)** Volcano plot representing gene expression in MSC-EV vs CDC-EV-treated mice. Genes with significant and higher than two-fold down- (green) and up-regulation (red) are referenced. ^*^ p<0.05, ^**^p<0.01, ^***^p<0.001. Bar graphs represent mean (±SEM).

Next, we decided to test the impact of CDC-EVs *in vivo*. In mice with a xenograft fibrosarcoma, both systemic and local treatment with human- or rat-CDC-EVs (Figure 2A, B) was associated with ∼2.4-fold decrease (p<0.01 and p<0.05 for human and rat, respectively) of tumor growth compared with phosphate-buffered saline (PBS) injected mice (Figure 2C–2F). Mean tumor weight was 1.5±0.3 gr and the proportion of mice with a tumor weight > 1.5 gr was 62.5% in the PBS group compared with 16.6% in the rat-CDC-EV-treated animals (p<0.05; Figure 2G). Decreased tumor growth was associated with 2-fold reduction of tumor cell proliferation measured by expression of Ki67 (p<0.001) and 1.5-fold increase of apoptosis based on terminal deoxynucleotidyl transferase dUTP nick end labeling (p<0.05) in human CDC-EV vs PBS mice (Figure 3A, B).

To further probe the mechanisms underlying the observed anti-cancer effects of CDC-EVs, we performed genomic and proteomic studies of the tumor using the same arrays as for *in vitro* studies. Interestingly the pattern of gene expression differences between control mice and both CDC-EV groups (systemic human (Figure 3C) and local rat (Supplementary Figure 2Ai)) were similar, indicating a clear negative effect of CDC-EVs regardless of the EV species of origin or the delivery method. A generalized downregulation of cancer drug target genes was observed in the CDC-EV groups which reached statistical significance in 38% of the transcripts quantified (Supplementary Figure 2B). Most of the downregulated genes were growth factors and their receptors, and transcription factors (Figure 3D). Comparing the *in vivo* and *in vitro* results, we observed that, while downregulation of ERBB2/ERBB3, MDM4, IGF1R or IRF5 may be direct effects of CDC-EVs on the cancer cells, many others (such as a reduced expression of HIF1A, CTSL, TOP2A or PLK2) are indirect, host cell-mediated effects with a final negative impact on tumor growth.

**Figure 4:**
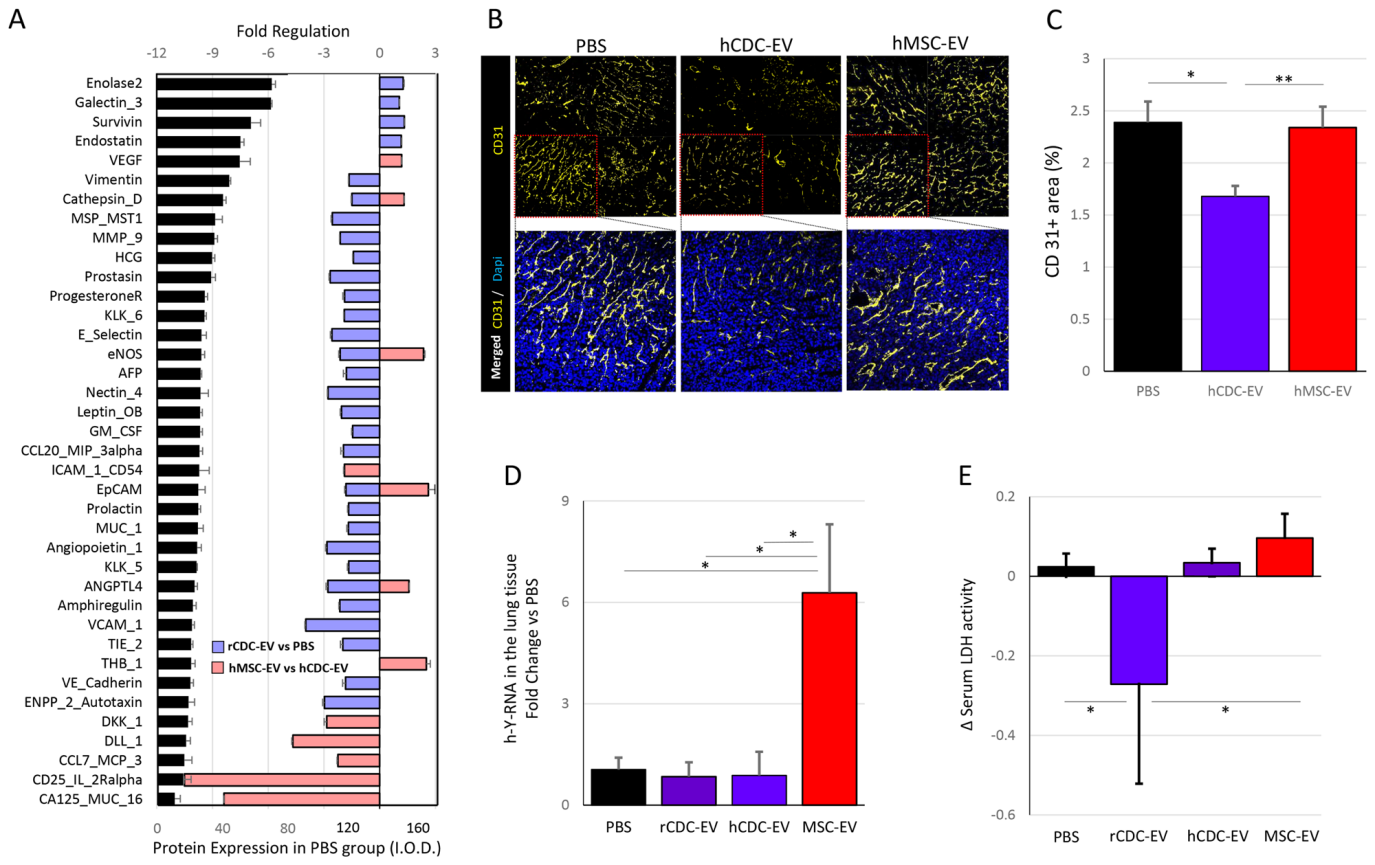
Tumor vascularization and lung metastases are attenuated by the treatment with CDC-EVs in mice with fibrosarcoma **(A)** Cancer-related proteins’ expression at the local tumor in the PBS (n=6) injected mice (black bars). Colored bars show the fold-regulation in proteins’ level in the CDC-EV (n=6) vs PBS-injected (lilac) and MSC-EV (n=4) vs human CDC-EV-treated mice (pink). Only proteins with significant (p<0.05) differences between groups are shown. **(B)** Immunostaining for endothelial cell marker CD31 shows lower tumor vascularization in the CDC-EVs-treated mice (n=5) compared with other control groups (PBS, n=5 and MSC-EVs, n=6). Each red framed square in the upper row of images corresponds to one animal, thus sections from four animals per group are jointly shown in the upper pictures. **(C)** Graph showing differences in expression of CD-31 in CDC-EV treated mice and both control groups. **(D)** Presence of HT1080 fibrosarcoma cells in the whole mice lung lysates was analyzed by measurement of the expression of the human Y-RNA fragment normalized for the expression of mice U6 with q-PCR. The results reveal higher presence of cancer cells in the lungs of MSC-EV-treated mice (n=6) compared to the remaining groups (PBS, n=8; hCDC-EV, n=5; rCDC-EV, n=4). **(E)** Changes in serum LDH activity were measured at days 18 and 25, showing significant decrease in the rCDC-EV-treated mice (n=4) compared to PBS (n=8) and MSC-EV (n=5) groups. The number of mice in the hCDC-EV was 4. ^*^ p <0.05, ^**^ p<0.01. Bar graphs represent mean (±SEM).

Differences in tumor protein levels were in parallel with the previously-reported gene expression results. Of 84 analyzed proteins, 30 showed significant modulation with CDC-EV treatment compared with PBS treated animals (Figure 4A). In 87% of cases, the proteins belonged to pathways related to local tumor progression, metastasis and/or angiogenesis (i.e. cathepsin D, MMP-9, eNOS, Leptin, ANGPTL4, autotaxin). We further looked at the endothelial cell marker CD31 (Figure 4B) and observed significantly lower vascularization of human CDC-EV treated mice tumors compared with PBS group (p<0.05; Figure 4C).

### MSC-EVs increase metastatic spread of cancer cells with related increased tumor vascularization compared with CDC-EV

Bone marrow-derived mesenchymal stem cells (MSC) are commonly used in regenerative medicine trials, but their safety in cancer is controversial [12–14]. Given this concern we used MSC-derived EVs (MSC-EV) as a second comparator group for the CDC-EV treated group of mice (Figure 2A). Unlike CDC-EV associated decrease of the tumor volume, MSC-EV treated mice did not present significant differences in the external tumor growth compared with PBS (Figure 2C, E, F), and animals in the MSC-EV group had higher tumor weight compared with rat-CDC-EV treated mice (p<0.05; Figure 2G).

Although tumor cell proliferation was lower in the MSC-EV treated mice than in the PBS group (p<0.01), it was higher than in CDC-EV treated animals (p<0.05; Figure 3A, B). No significant effect was observed on apoptosis (Figure 3A, B). Analysis of the gene expression pattern revealed some directionally-opposite effects in MSC-EV vs CDC-EV treated mice, as compared to the differences between CDC-EV and PBS animals (Figure 3E). In the latter case, most of the genes were downregulated, but MSC-EV mice had higher expression levels of cathepsins (CTSS and CTSD) and growth factors such as ERBB4, ERBB2, FIGF, EGFR compared to CDC-EV treated mice (Supplementary Figure 2C). Curiously, telomerase (TERT) expression was also 1.6-fold higher (p<0.01) in the MSC-EV vs CDC-EV groups. Other differences such as markedly upregulated expression of PTGS2 (3.8-fold; p<0.00001) and HDAC11 (3.1-fold; p<0.001) in MSC-EV vs CDC-EV animals, may reflect higher pro-inflammatory properties of the former particles.

Similarly to the gene expression, differences in tumor protein levels in MSC-EV treated mice were opposite to those observed in CDC-EV injected animals for roughly half the proteins probed (Figure 4A). While downregulated in CDC-EV vs PBS groups, levels of cathepsin D, eNOS, EpCAM and ANGPTL4 were up-regulated in MSC-EV vs CDC-EV treated mice. All promote tumor development by increased angiogenesis (VEGF expression was also significantly higher in the MSC-EV group) and/or invasiveness of tumor cells [15–19]. We further confirmed higher vascularization of the tumor in MSC-EV compared with the CDC-EV group of mice by CD31 staining (p<0.01; Figure 4B, C).

To analyze the metastatic spread of cancer cells, we searched for human Y-RNA fragments in mouse lung tissue with q-PCR. With high-moderate expression (Ct values <30 for almost all animals; Supplementary Figure 3B), we found 6-fold higher levels of HT1080 fibrosarcoma cells in the lungs of MSC-EV treated mice vs PBS or CDC-EV groups (p<0.05 for all comparisons; Figure 4D). Although not specific, we analyzed changes in serum levels of lactate dehydrogenase (LDH), in an attempt to assess the systemic impact of the cancer [20, 21]. High LDH levels are associated with an increased risk of death from prostate, pulmonary, colorectal, gastro-esophageal, gynecological and hematological cancers [22] and changes in LDH levels during treatment may also predict overall survival in patients with metastatic cancer [23]. We found a moderate increase of serum LDH levels in MSC-EV treated mice, unlike the marked decrease observed in the rat-CDC-EV treated group (p<0.05; Figure 4E).

### CDC-EV decrease spontaneous leukemia-related mortality in old rats

**Figure 5:**
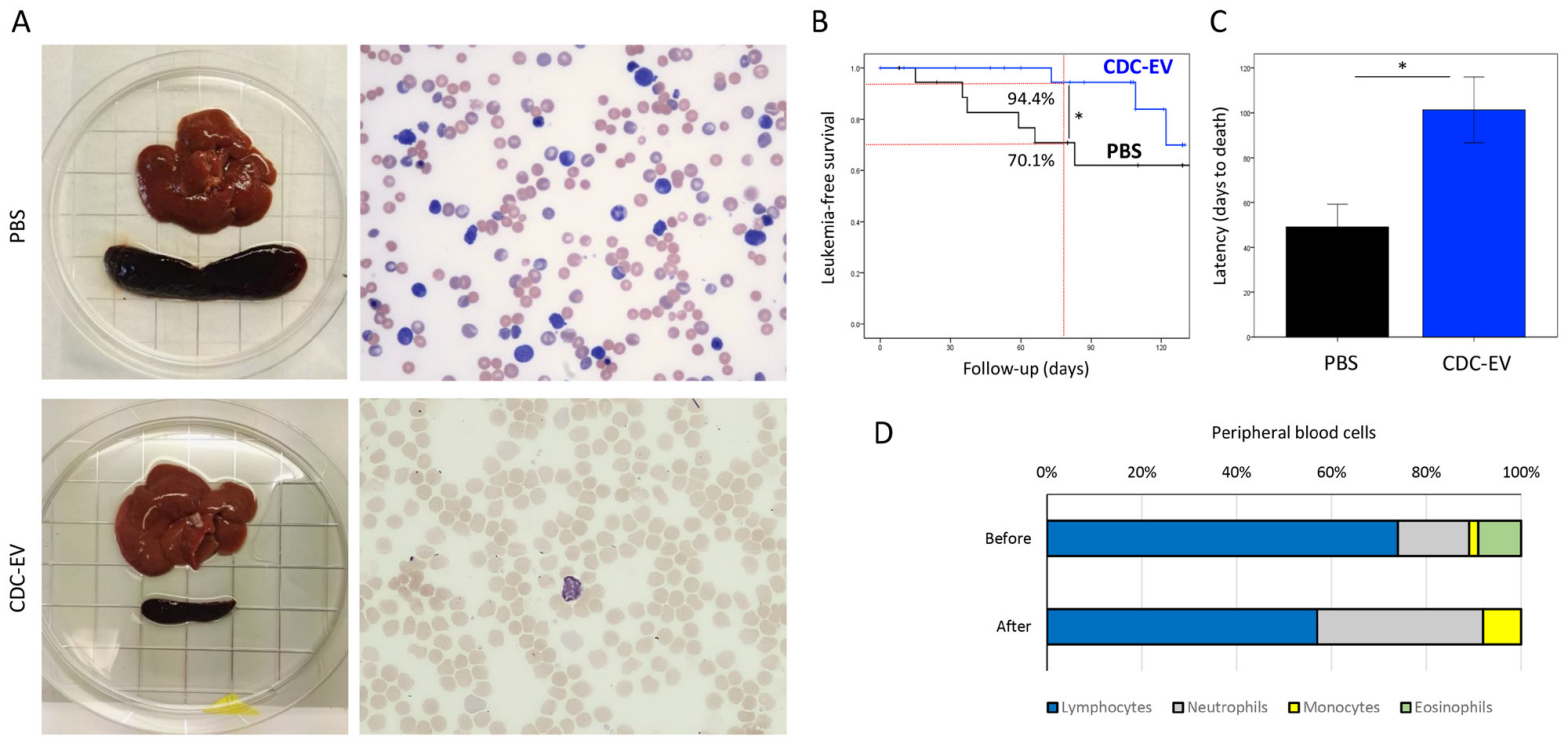
CDC-EVs decrease the incidence of spontaneous leukemia and increase the survival in old rats **(A)** Representative images showing marked splenomegaly and increased number of immature, enlarged lymphoid cells in the blood smear of a rat from PBS group in contrast with a normal spleen and blood smear in a CDC-EV-treated rat. **(B)** Kaplan-Meier leukemia-free survival curves in CDC-EV (n=24) and PBS (n=20) rats. **(C)** The latency to leukemia-related mortality was doubled in CDC-EV rats. **(D)** Changes in proportional distribution of white blood cells in peripheral blood in a leukemic CDC-EV rat one-week after administration of EVs. ^*^ p<0.05. Bar graph represent mean (±SD).

In the process of studying the rejuvenating effects of CDC-EVs in old rats, we serendipitously noted an effect of CDC-EVs on spontaneous acute leukemia, which is known to be prevalent and fatal in senescent rats [24]. Animals treated with rat-CDC-EVs less frequently developed clinically overt acute lymphocyte leukemia (ALL, characterized by jaundice, ∼4-fold increase of spleen size and abnormal blood counts), than did PBS rats (12.5% vs 30%, p=n.s.) (Figure 5A). Mean leukemia-free survival also increased from 107±11 days in the PBS group to 124±4 days in rats treated with CDC-EVs (p<0.05; Figure 5B). The latency for the development of advanced disease and death was increased two-fold in the CDC-EV group vs PBS (p<0.05; Figure 5C).

Only 3 of 24 rats in the CDC-EV arm developed clinical ALL. In one of the rats, once the diagnosis was confirmed by blood counting, an additional, double dose of rat-CDC-EVs was administered to evaluate the effect on cancer cells. After one week, the total number of white blood cells decreased from 82.6x10^3^/µL to 34.6x10^3^/µL, the absolute number of lymphocytes from 61124/ µL to 19722/µL, the 12390/µL neutrophils remained almost unchanged and monocytes increased from 1652/µL to 2768/µL, resulting in a change of the proportional distribution of the blood cells (Figure 5D), suggestive of an anti-leukemic effect.

### Differential miR signature of EVs as a potential contributor to the anti-cancer effects of the CDCs

EVs carry and transfer a diverse cargo including proteins, lipids and nucleic acids. MiRs, small regulatory RNA molecules stably transported by EVs, influence the expression of >60% of human protein-coding genes [25]. Recently they have been demonstrated to affect the hallmarks of cancer, including sustaining proliferative signaling, evading growth suppressors, resisting cell death, activating invasion and metastasis, and inducing angiogenesis [26, 27].

**Figure 6:**
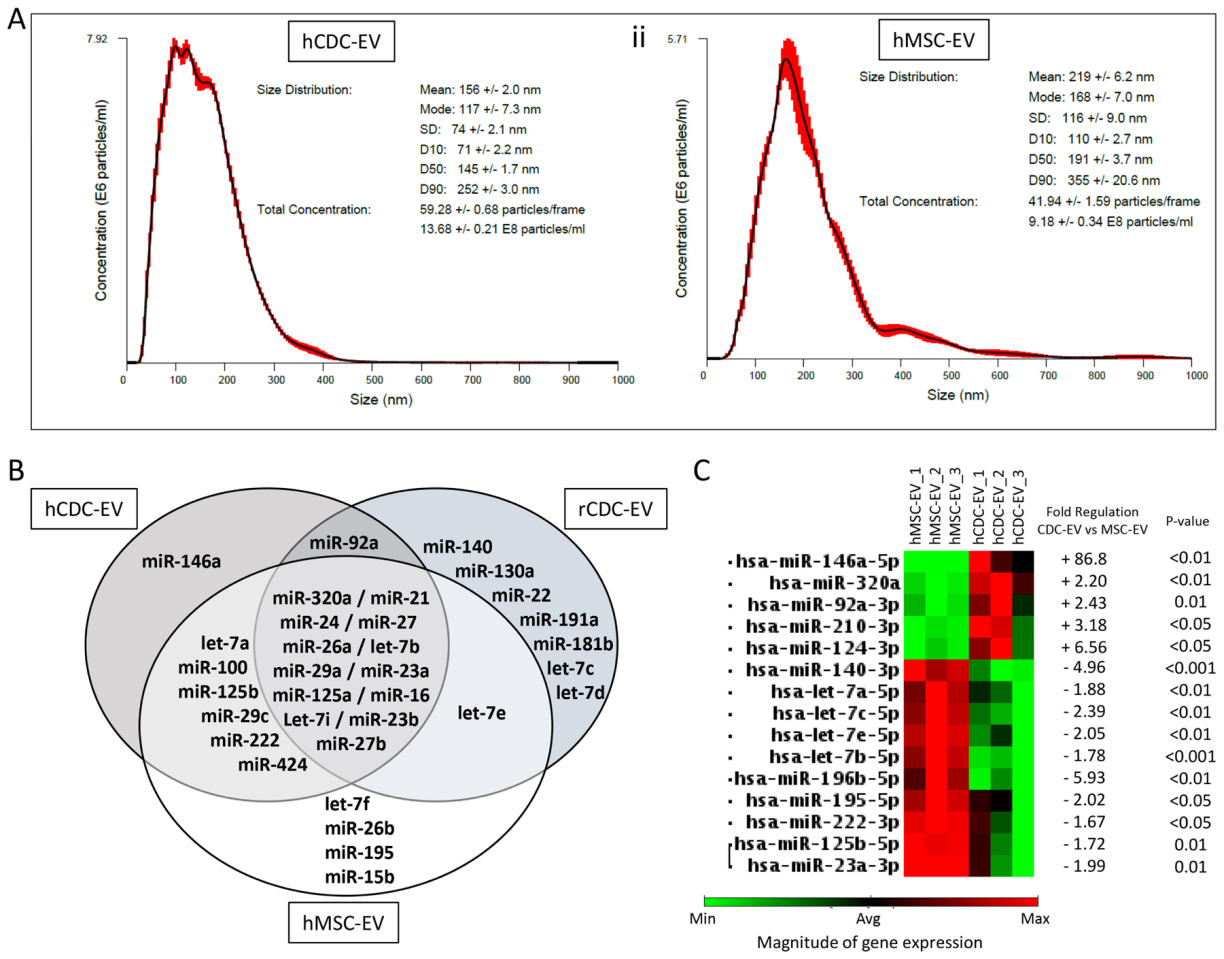
Differences between EVs secreted by the cardiosphere-derived cells (CDCs), and bone marrow-derived mesenchymial stem cells (MSCs) **(A)** Size distribution and EVs number secreted by the CDCs and MSCs measured by Nanoparticles Tracking Analysis. **(B)** Distribution of most abundant micro-RNA (miRs) in human- (hCDC-EV), rat-CDC-EV (rCDC-EV) and human MSC-EVs analyzed with an array. **(C)** Cluster map with differentially expressed miRs (abundant and non-abundant) in human CDC-EVs vs MSC-EVs.

Based on this evidence and the oncogenic differences we observed between CDC- and MSC-EVs, we focused on the EVs’ miR cargo. First, we observed that the same passage and number of initially-plated cells, obtained from human donors of similar age, differ in their EV production: CDCs secreted ∼50% more EVs than did MSCs, and the mean size of the particles was ∼50 nm smaller (Figure 6A). This may be related to higher secretion of exosomes, which are the smallest EVs, by CDCs. Next, in comparing EV miRs (Figure 6B), we observed that miR-146a was exclusive for human CDC-EVs and miR-92a was exclusive for human and rat CDC-EVs among the most abundant miRs. Globally (among abundant and non-abundant miRs), miR-146a was 87-fold up-regulated in human CDC-EVs compared with MSC-EVs (p<0.01; Figure 6C) and only 6.2-fold higher compared with rat-CDC-EVs. Other miRs which were more abundant in CDC-EVs vs MSC-EVs included miR-124, miR-210, miR-92 and miR-320.

## DISCUSSION

This study shed light on the anti-oncogenic nature of the heart microenvironment. We demonstrated a reduction of proliferation and an increase of programmed death of tumor cells, together with a prolongation of host survival in two different cancer types and different rodent species, associated with the use of human- and rat-CDC-EVs. The parent CDCs have been tested in different pre-clinical and clinical studies for therapeutic applications unrelated to cancer; they have been demonstrated to have many favorable effects and no safety issues to date [7-9, 28, 29]. In the field of oncology, a class of drugs that efficiently eliminates all cancer cells with no or minimal toxicity for normal cells is still not available. So, the relevance of our finding is that CDC-EVs may constitute a novel, non-toxic anticancer treatment approach.

We observed that the reduction of tumor growth in CDC-EV treated animals was associated with a wide, local modulation of genomic and proteomic profiles, consistent with the diversity of bioactive components within EVs. Underlying the inhibition of tumor progression were CDC-EV-induced down-regulation of growth factors and their receptors, decreased levels of transcription factors, and a marked anti-angiogenic effect. The last effect has implications not only for the local development of the cancer but for metastatic potential as well [30, 31]. The amplification (in terms of a higher number of modulated genes and proteins) of the CDC-EV induced anticancer effect *in vivo* compared with our *in vitro* studies, suggests that part of the effect is mediated indirectly, probably through reconditioning of different host cells [32]. Tumor microenvironment is known to be essential for sustained cancer growth, invasion and metastasis [6] and tumor stromal cells have been proposed as an attractive therapeutic target [33]. CDCs were demonstrated to modulate fibroblasts [32] and macrophages [28], both cell types with relevance to the tumor microenvironment [34, 35]. CDCs were also demonstrated to reduce tissue myofibroblast infiltration [29], which release metalloproteinases (MMP) and lead to extracellular matrix (ECM) remodeling and the liberation of growth factors embedded in the ECM, tumor growth, local invasion and vascularization. Activated tumor-associated macrophages secrete G-CSF, IL-6 and VEGF, promoting angiogenesis and creating an inflammatory niche [11, 36, 37]. We observed reduced levels of many of these proteins in the tumors of CDC-EV treated mice compared with controls.

In studying CDC-EVs, we have noted a relationship between anti-aging and anti-cancer effects. CDC-EVs from young donors have local and systemic rejuvenating properties [38, 39]. A potential link to anti-tumor effects is perhaps not surprising. The rejuvenating effects of miR-146 on fibroblasts are associated with inhibition of IL-6 expression [40], a key mediator of the senescence-associated secretory phenotype [41]. Meanwhile, miR-146 may appears to act as a tumor suppressor for many solid and hematological malignancies [42]. Although the mechanism of miR-146-mediated tumor suppression is still unclear, EGF-R was identified as a target of this miR [42]. Increased miR-146 and a subsequent decline of EGF-R expression are associated with decreased proliferation, and inhibited invasion and migration of tumor cells in breast, pancreatic and gastric cancer [43, 44]. Mouse miR-146 knockout models strongly support a role for miR-146 as a tumor suppressor for myelo-lymphoid cells [45]. Both IL-6 and EGF-R were negatively modulated by CDC-EVs in our study, supporting the simplistic idea that CDC-EVs may act as a source of miR-146 as one possible anti-oncogenic mechanism. Another miR similarly abundant in human- and rat-CDC-EVs, and significantly higher compared with MSC-EVs, was miR-92, a member of the miR-17-92 cluster, and an important regulator of cancer and aging [46]. HIF-1α was recently identified as a target of the cluster [47] and in fact, we detected a significant downregulation of this gene both with human- and rat-CDC-EVs. Although the evidence suggests that cell type-specific responses are possible in response to miR-17-92, downregulation of miR-92a specifically triggers macrophage infiltration of the tumor stroma, promotes cell migration and decreases survival in breast cancer patients [48].

Contrary to CDC-EVs, the use of MSC-EVs was associated with greater lung metastasis. Our results are not novel in this sense as there is no consensus regarding the safety profile of MSCs used in regenerative medicine. MSCs may exert pro-tumorigenic effects by inducing immunosuppression, promoting angiogenesis and/or stimulating epithelial-to-mesenchymal transition [12]. We observed a significant up-regulation of genes implicated in inflammation, and proteins such as cathepsin D, eNOS, EpCAM and ANGPTL4 in MSC-EV vs CDC-EV treated animals. All these proteins have been described as associated with increased growth, invasiveness, angiogenesis and metastasis in different studies [15–19]. Moreover, miR-222/223 enriched in the MSC-EVs vs CDC-EVs in our study, was implicated in inducing dormancy and prolonged survival of breast cancer cells together with increased drug resistance [49]. Our results highlight the need for more research to guarantee the safe use of MSCs in other fields where these cells may exert potential benefits.

### Limitation

The main limitation of our study is the use of uncommon cancer models. Fibrosarcoma HT1080 cells, although extensively used in biomedical research, were first characterized more than forty years ago. Such a long-term storage of cells may have introduced unexpected genetic modifications and other changes in the cell line. Secondly, fibrosarcomas are rare tumors, which only comprise ∼10% of all sarcomas in recent series. Likewise, the ALL we evaluated in this study, although common in old Fisher344 rats, is rare in humans, most closely resembling natural killer large granular lymphocyte leukemia [24]. However, the fact that very common cancer-related pathways were beneficially modulated by CDC-EVs in our study, together with an anti-tumorigenic effect on spontaneous cancer incidence and survival, in two different species, support the possible generalizability of the observed effects. Nevertheless, this hypothesis should be confirmed in other, more common types of human cancer.

Second, the study lacks a clear mechanistic explanation for the anti-cancer effect of CDC-EVs. We generated a hypothesis based on miR cargo of the CDC-EVs; however, a role for other EV contents was not excluded. Moreover, a polyethylene glycol-based method to isolate EVs used in our study, although accepted [50], does not guarantee a total purification of the vesicles from the medium.

Third, our findings demonstrate an anti-oncogenic effect of exogenous heart-derived EVs, but the notion that naturally-secreted heart-derived EVs suppress neoplasia *in situ* remains conjectural.

## CONCLUSIONS

This study presents the first evidence that ties together the long-recognized enigma of the “heart immunity to cancer” with an antioncogenic effect of heart-derived EVs. These findings open a new therapeutic opportunity for anti-oncogenic EVs.

## MATERIALS AND METHODS

### Animal models

Two different animal models were used: human xenograft fibrosarcoma in nude athymic Foxn1nu mice, and spontaneous ALL in 2-year old Fisher 344 rats. The ALL, also denoted as large granular lymphocyte (LGL) leukemia, is one of the leading causes of death in old F344 rats [51]. The phenotype of the leukemic cells resembles that of human NK-LGL leukemia [24]. All animal procedures were conducted in accordance with humane animal care standards outlined in the NIH Guide for the Care and Use of Experimental Animals and were approved by the Cedars-Sinai Animal Care and Use Committee.

### Cell lines

HT1080 human fibrosarcoma cells were purchased from ATCC. Human bone marrow-derived MSCs were purchased from Lonza. Cells were cultured per manufacturer’s instructions. CDCs from human donors and rat hearts were derived in our lab, as described [52].

### Extracellular vesicles isolation and characterization

EVs were harvested from serum-free media conditioned by MSCs or CDCs. The procedure is fully described in the supplementary file.

### MicroRNA array analysis

To characterize the microRNA (miR) cargo of CDC- and MSC-derived EVs, miR was extracted using the miRNeasy kit (Qiagen) and analyzed with MiScript array with a total of 88 genes (Qiagen), according to manufacturer’s instructions.

### *In vitro* studies

HT1080 were incubated in SF medium alone or SF containing resuspended CDC-EVs (1 mg of EV-protein per 10^6^ cells; Supplementary Figure 1A). After 96 or 120 hours, the cells were harvested, washed in PBS and used for various assays. See supplementary data for detailed information on telomerase activity, invasion and adhesion assays, PCR array, and proteome analysis performed *in vitro*.

### *In vivo* studies

To create xenograft tumors, 10^6^ HT1080 cells, resuspended in PBS, were injected subcutaneously into the right and left flanks of nude athymic mice. The animals were then subjected to either of two different protocols based on the EV-delivery method and the dose.

In the systemic-delivery protocol using intraperitoneal (i.p.) administration 24-hours after HT1080 cell injection, mice were randomly allocated among three groups: human-CDC-EVs (n=6) (CDC-EV hereinafter, unless host specified), human-MSC-EVs (n=8), or PBS alone (n=9). EVs (1mg protein) were resuspended in 1mL of PBS or 1mL PBS alone was used and repeated after 2 weeks. In the local-delivery protocol, using peri-tumoral subcutaneous (s.c.) administration of treatment 24-hours after HT 1080 cell injection, mice were randomly allocated to either of two groups: rat-CDC-EVs (n=6) or PBS alone (n=6). EVs (2mg protein) were resuspended in 1mL of PBS, or 1mL PBS alone, was injected locally and repeated on a weekly basis.

In the rat model of spontaneous ALL, after initial functional evaluation, a total of 44 animals (males and females) were allocated in two groups, ensuring similar baseline characteristics. Twenty-four rats received a dose of 7 µg-EV protein/gr body weight of rat-CDC-EV via systemic intra-arterial (i.a.) injection. The remaining (n=20) rats, in the control group, received i.a. PBS monthly during the 4-month follow-up period. The diagnosis of ALL was established when the clinical picture was associated with either typical histopathological findings in the spleen and/or an abnormal peripheral blood test. Clinically, affected rats exhibited progressive decreases of exercise capacity, weight loss, pale eyes and jaundice. Splenomegaly was a constant finding in these rats. Histological findings included diffuse infiltration of the splenic red pulp with the neoplastic LGL; peripheral blood was characterized by marked leukocytosis (> 50×103/µL, upper limit of normal 11×103/µL) with atypical lymphocytosis (LGL). Regenerative anemia, thrombocytopenia and abnormal liver function tests were common findings, and death usually occurred within 2-3 weeks of the first clinical signs.

### Statistical analysis

All results are presented as mean ± SEM or percentages, for continuous and categorical variables, respectively. Significance of differences was assessed by Student t test or 1-way ANOVA in cases of multiple groups if the distribution of the variable was normal; otherwise, the Mann-Whitney or Kruskal-Wallis tests were used. Tumor volume data were tested across treatment groups with mixed-model regression to account for the repeated measures within each animal. Post-hoc testing was adjusted for multiple comparisons (Tukey). Data was log-transformed prior to analysis and residuals were inspected to confirm data met assumptions necessary for parametric assessment. For survival analysis Breslow-Wilcoxon test was applied to compare leukemia-free survival curves. All probability values reported are 2-sided, with p<0.05 considered significant. IBM SPSS Statistics 20 and SAS v9.4 were used for all analyses. For *in vitro* studies the lowest number of replicates per experiment was three.

## SUPPLEMENTARY MATERIALS FIGURES



## Abbreviations

ALL: acute lymphocyte leukemia
ANGPTL4: angiopoietin-like 4
CA125: cancer Antigen 125
CDCs: cardiosphere-derived cells (heart progenitor cells)
CDC-EVs: extracellular vesicles secreted by the cardio sphere-derived cells
CTSL: cathepsin L
dUTP: terminal deoxynucleotidyl transferase
EGFR: epidermal growth factor receptor
eNOS: endothelial nitric oxide synthases
ERBB2, ERBB3, ERBB4: epidermal growth factor receptors
EpCAM: epithelial cell adhesion molecule
EVs: extracellular vesicles
FIGF: C-fos-induced growth factor
HDAC11: histone deacetylase 11
HIF1A: hypoxia-inducible factor 1-alpha
i.a.: intra-arterial injections
IGF1R: insulin-like growth factor 1 receptor
IL-6: interleukin 6
i.p.: intra-peritoneal injections
IRF5: interferon regulatory factor 5
LDH: lactate dehydrogenase
LGL: large granular lymphocyte leukemia
MiR: micro-RNA
MMP-9: matrix metallopeptidase 9
MSC-EVs: extracellular vesicles secreted by bone-marrow derived mesenchymal stem cells
NK: Natural killer cells
PBS: phosphate-buffered saline
PDGF-AA: platelet-derived growth factor-AA
PLK2: serine/threonine-protein kinase PLK2
PTGS2: prostaglandin-endoperoxide synthase 2, cyclooxygenase-2
s.c.: subcutaneous injections
SF: serum-free medium
TERT: telomerase reverse transcriptase
TOP2A: DNA topoisomerase 2-alpha
VEGF: Vascular endothelial growth factor
