# Supplementary material for: Harnessing the heart’s resistance to malignant tumors: cardiac-derived extracellular vesicles decrease fibrosarcoma growth and leukemia-related mortality in rodents
Source: Oncotarget. 2017 Aug 24;8(59):99624–36. doi: 10.18632/oncotarget.20454 (PMC5725120; doi:10.18632/oncotarget.20454)
Supplement: Supplementary file 1 [file oncotarget-08-99624-s001.pdf]

Harnessing the heart's resistance to malignant tumors: cardiac-derived extracellular vesicles decrease fibrosarcoma growth and leukemia-related mortality in rodents

SUPPLEMENTARY MATERIALS

METHODS

Heart cell isolation and culturing

When minced heart tissue is grown in primary culture, it spontaneously gives rise to monolayers of cardiac stromal cells and progenitor cells known as explant-derived cells (EDCs). Such EDCs are the precursors to cardiospheres and cardiosphere-derived cells (CDCs). Methods for EDC and CDC isolation and culture were described. In brief, myocardial biopsies from healthy hearts of deceased tissue donors (for human tissue) were minced into small fragments, digested with collagenase, and cultured on fibronectin-coated dishes. EDCs grow spontaneously from the tissue fragments and reach confluence by 2–3 weeks, at which time they are harvested using 0.25% trypsin (GIBCO), purified from tissue and cell debris and re-plated. EDCs were cultured in suspension on 20 µg/ml poly d-lysine (BD Biosciences) to form self-aggregating cardiospheres. CDCs were obtained by seeding cardiospheres onto fibronectin-coated dishes and passaged 2-4 times. Cultures were maintained in 5% CO2 at 37°C, using IMDM basic medium (GIBCO) supplemented with 20% FBS (Hyclone), 1% penicillin/streptomycin, and 0.1 ml 2-mercaptoethanol. All protocols were approved by the institutional review board for human and animal subjects research.

Isolation and characterization of extracellular vesicles

Extracellular vesicles (EVs) were harvested from serum-free condition media conditioned for 15 days by passage 3 CDCs and bone marrow-derived mesenchymal stem cells (MSCs). Media was then subjected to two successive centrifugation steps to remove cellular debris: 2,000xg for 20 min and 10,000xg for 30 min. The resulting supernatant was precipitated by polyethylene glycol (ExoQuickTC), which yields high quantities of purified exosomes (), followed by overnight incubation at 4ºC. EVs were then isolated by centrifugation at 2,000xg for 30 min, resuspended in phosphate buffered saline (PBS) and quantified for particle concentration and size using the LM10-HS system (NanoSight), and protein concentration (BQuick StartTM).

In vitro studies

HT1080 cells were counted manually using the Neubauer Ruled hemocytometer and reconfirmed with a TC20™ Automated Cell Counter. Viability was checked by trypan blue staining. Equal number of viable cells were plated in 6 or 12-well plates; after an initial 24-hour stabilization period in minimal essential medium, Eagle's minimal essential medium, and Dulbecco's Modified Eagle's Medium (Thermo Fisher Scientific) fetal-bovine-serum (10%) supplemented medium, the cells were washed with PBS and then incubated with serum-free medium (SF) alone or containing resuspended CDC-EVs (1 mg of EV-protein per 10^6 cells; Supplementary Figure 1A). After 96 or 120 hours the cells were harvested, washed in PBS and used for various assays.

In vivo studies

To measure the tumor growth in the mice fibrosarcoma model, the two longest perpendicular axes in the x/y plane and the height (z) of each xenograft tumor were measured periodically, using a digital Vernier caliper. The tumor volume was calculated according to equation $Vol = a \times b^x z$ (z was assumed 1mm when the tumor height was inaccessible for measuring at initial stages). Food-water consumption and body weight were also measured, to monitor for cachexia. Blood samples were obtained on days 18 and 25. The animals were followed-up for 30 days, euthanized, and tumors and lungs were harvested for further analysis.

Invasion assay

Invasion assays were done using a Cytoselect 24 well Cell Invasion Assay (colorimetric) kit (Cell Biolabs). An aliquot of the suspension was added to inserts coated with a uniform layer of reconstituted basement membrane proteins (ECMatrix). Minimal Essential Medium containing 10 % FBS was added to the lower chamber in which the coated inserts were placed. At 48 hours of incubation, the cells that had not invaded the membrane were washed away, and the invasive cells were stained with Cell stain solution. The dye from the stained cells was then extracted, and the color intensity was measured at OD 560 nm using a microplate reader.

Adhesion assay

Cell adhesion potential was tested using CytoSelect™ 48-well Cell Adhesion Assay (ECM Array,

Colorimetric Format). An equal number of cells was added to each Fibrinogen-coated well. After 1.5 hours of incubation, the plate was rinsed with PBS to discard the unattached cells, and the attached cells were stained with a cell stain solution. After extracting the dye from attached cells, the color intensity was measured at OD 560 nm using a microplate reader.

Telomerase activity assay

Telomerase activity was determined using TeloTAGGG Telomerase PCR ELISAPLUS kit (Sigma-Aldrich). Cells were lysed with the mixture of lysis reagent and HaltTM protease inhibitor (Thermo Fisher Scientific) and checked for protein concentration using (Bio-Rad Quick StartTM). The cell extract was heated for 10 min at +85°C to create negative controls for each sample. One ug of total protein was used from each sample for combined primer elongation (telomeric repeat amplification protocol [TRAP reaction])/amplification (PCR) reaction. PCR products were then denatured and hybridized with digoxigenin (DIG)-labeled detection probes which is specific for telomeric repeats. The biotin-labeled primer was then used to immobilize the resulting product to a streptavidin-coated microplate, which was then detected with an anti-digoxigenin antibody conjugated with horseradish peroxidase and TMB substrate. The plate was then read twice with a microplate reader at OD 450 nm.

RT-PCR Array

RNA was extracted from Ht1080 cells using RNeasy Plus mini kit (QIAGEN) and from tumor tissue using RNeasy plus universal kit (QIAGEN). For further purification of RNA, the RNase-Free DNase set (Qiagen) was used, and the RNA integrity was assessed by Nano drop. cDNA was generated from the purified RNA using RT2 first strand kit. PCR was performed on Applied bio system 96 well fast block cycler using RT2 Profiler PCR Array human cancer drug target and RT2 Real-Time SYBR Green PCR Master Mix (Qiagen). The array evaluated the expression of 84 cancer marker genes and five housekeeping genes for data normalization. Gene expression was then amplified over the course of 40 cycles and analyzed by ddCt method.

Proteome profiler assay

Tumor tissues were lysed using a mixture of RIPA lysis extraction buffer and HaltTM protease inhibitor (Thermo Fisher Scientific), and cells were lysed using proteome profiler lysis buffer 17. Lysed cells and tumor tissue were incubated overnight with Human Phospho-Kinase Array nitrocellulose membranes which were spotted with different capture and control antibodies. Unbounded proteins were washed away, and the array was then incubated with a cocktail of biotinylated detection antibodies. After application of Streptavidin-HRP and chemiluminescent detection reagents, a signal was produced at each capture spot based on the amount of bounded phosphorylated protein. The average signal (pixel density) of the pair of duplicate spots representing each protein was determined after subtracting an averaged background signal.

LDH assay

Serum for LDH assay use was obtained from blood samples collected on day 18 and 25 from treated and untreated mice. After adding the master mix to the biological samples, the LDH activity was measured when LDH reduced NAD to NADH, which then interacted with a particular probe to produce a color. The output was then measured every 3 minutes at OD 450 on a light protected kinetic microplate reader at 37°C for 30 minutes.

Inmmunostaining

Tissue slices were washed with PBS and fixed with 4% paraformaldehyde followed by blocking solution (Dako). Slices were incubated overnight at 4°C with the primary antibody. Ki-67 and CD-31 antibodies were purchased from Abcam. Slices were washed 3 times with PBS and incubated with a secondary antibody of the appropriate species and *in situ* cell death detection kit, fluorescein (Roche). All slides were counterstained for DAPI (Sigma). Five to 10 images per slide were imaged at x20 magnification using a confocal laser microscope and analyzed using Image-J software.

A

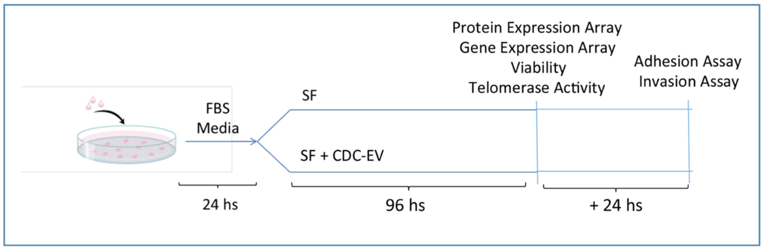

B

	Fold Regulation CDC-EV vs SF	p-value
PARP1	-1.92	0.003653
HDAC6	-1.82	0.015703
TXNRD1	-1.78	0.028951
ERBB3	-1.74	0.007890
MDM4	-1.6	0.027694
CDK5	-1.59	0.026650
AURKB	-1.55	0.021451
HDAC1	-1.54	0.026421
PLK1	-1.43	0.001768
PLK2	1.19	0.022510
PLK4	1.28	0.005505
TOP2B	1.32	0.016807
PIK3C2A	1.36	0.029868
RHOA	1.51	0.028415
HIF1A	1.52	0.007470
HSP90B1	1.53	0.022042
PGR	1.58	0.025435
PIK3C3	1.6	0.004922
PIK3CA	1.8	0.011178

Supplementary Figure 1: (A) Protocol used for the *in vitro* studies. **(B)** NCl-H23 neuroblastoma cells were less viable after priming them with extracellular vesicles secreted by the CDC-EVs vs SF (panel i). Invasion and adhesion were not significantly affected (panels ii and iii, respectively). **(C)** The complete list of significantly modulated genes after priming HT1080 fibrosarcoma cells with CDC-EVs compared to culture them in SF alone. $^{*}p<0.05$. Bar graphs represent mean (±SEM).

A i

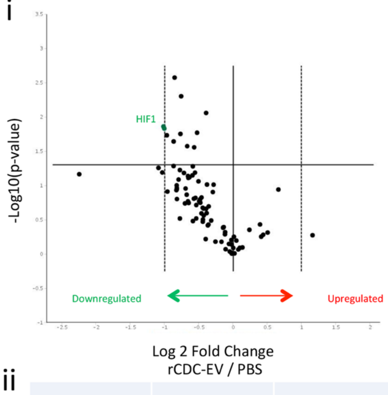

ii

Symbol	Fold Regulation	P-Value
HIF1A	-2.02297	0.013793
CTSL	-1.6916	0.005007
ERBB3	-1.80793	0.002675
ESR2	-1.43972	0.017031
FIGF	-1.48242	0.027828
HDAC1	-1.47034	0.065084
HDAC11	-4.7495	0.068611
HDAC2	-1.82547	0.022834
IRF5	-1.59121	0.026775
MDM4	-1.70341	0.01771
PDGFRA	-2.12897	0.05542
PGR	-1.82667	0.052066
PIK3C2A	-1.64474	0.065696
PLK2	-1.95624	0.018533
PRKCA	-1.71303	0.059564
PRKCD	-1.50295	0.052593
TOP2A	-2.04848	0.064712

B

Symbol	Fold Regulation	P-Value
ESR1	5.344907	0.000703
ATF2	-2.26182	0.016032
CTSD	-2.20599	0.002652
CTSL	-2.49587	0.000362
EGFR	-2.9358	0.018862
ERBB2	-2.31896	0.000067
ERBB3	-3.18337	0.000297
FIGF	-2.97107	0.000722
HDAC1	-2.15631	0.004692
HIF1A	-2.46335	0.01053
IGF1R	-2.15186	0.000341
IRF5	-2.07154	0.000108
NRAS	-2.24988	0.01875
PGR	-2.27273	0.022818
PIK3C2A	-2.30325	0.011544
PRKCA	-3.04015	0.00897
PRKCB	-2.39305	0.019778
TNKS	-2.31325	0.047802
TOP2A	-2.53581	0.039343
TP53	-2.31146	0.049788
ABCC1	-1.6111	0.019556
AKT1	-1.60627	0.013215
AKT2	-1.73648	0.050027
CTSB	-1.60357	0.006862
GRB2	-1.96988	0.035335
HDAC2	-1.79588	0.010076
HDAC4	-1.87968	0.011918
HDAC6	-1.4407	0.019051
KRAS	-1.87504	0.030588
MDM4	-1.77688	0.003945
PLK2	-1.68516	0.021749
PLK3	-1.81691	0.008379
PRKCD	-1.72375	0.023054

C

Symbol	Fold Regulation	P-Value
PTGS2	3.840649	0.000001
ERBB4	3.409886	0.0004
PLK3	3.099962	0.022027
HDAC11	3.057121	0.000253
CTSS	2.542025	0.04584
RHOB	2.207766	0.008711
EGFR	1.835782	0.007086
ERBB2	1.800636	0.000565
TERT	1.646559	0.001055
FIGF	1.632188	0.061858
CTSD	1.619735	0.014168
IRF5	1.610349	0.069123
TP53	1.445963	0.002587
AURKC	1.436586	0.010078
MDM4	1.343562	0.001686
TNKS	1.29314	0.036987
HDAC7	1.29289	0.00012
PGR	1.265273	0.000015
MDM2	1.184841	0.017195
PRKCD	1.072644	0.008709
HRAS	-1.38729	0.000448
GSTP1	-1.39209	0.020681
KDR	-1.40814	0.061082
MTOR	-1.44672	0.0252
PRKCA	-1.51341	0.020307
CDC25A	-1.65525	0.032224
CDK5	-1.69791	0.000013
ESR2	-1.70752	0.053452
AURKB	-1.82288	0.014047
BIRC5	-2.04594	0.001261
PRKCB	-2.38835	0.051038

Supplementary Figure 2: Transcriptional changes at the tumor site in mice with fibrosarcoma. (Ai) Volcano plot representing gene expression in rat CDC-EV vs PBS-treated mice. HIF1 was the only gene with a significant and higher than two-fold down-regulation. No up-regulated genes were detected. **(Aii)** The complete list of significantly modulated genes in rat CDC-EV vs PBS groups. **(B)** The complete list of significantly modulated genes in human CDC-EV vs PBS groups. **(C)** The complete list of significantly modulated genes in human MSC-EV vs CDC-EV groups. Genes with borderline significance changes are included in the lists as well.

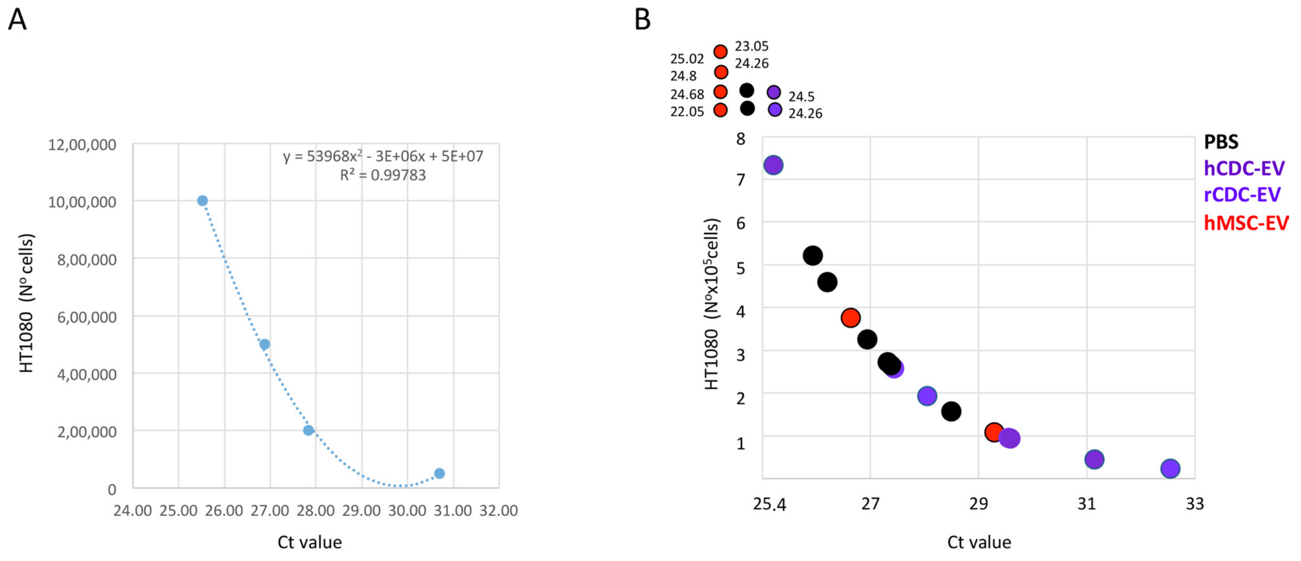

Supplementary Figure 3: Presence of HT1080 fibrosarcoma cells in lung tissue analyzed by measuring the expression of human Y-RNA fragment with q-PCR. **(A)** Standard curve was built using increasing numbers of pure HT1080 cells (range 10^3 to 10^6). **(B)** Plotted results correlating the number of HT1080 cells (Y-axis) with the Ct-values for human Y-RNA expression (X-axis) obtained in the lung tissue of different mice. In some cases, the results fell out of the lower Ct-limit of the standard curve, these are shown out of the plot and individual Ct values are referenced.
